# Biological clocks and physical functioning in monozygotic female twins

**DOI:** 10.1186/s12877-018-0775-6

**Published:** 2018-04-04

**Authors:** Elina Sillanpää, Eija K. Laakkonen, Elina Vaara, Taina Rantanen, Vuokko Kovanen, Sarianna Sipilä, Jaakko Kaprio, Miina Ollikainen

**Affiliations:** 1 Gerontology Research Center, Faculty of Sport and Health Sciences, University of Jyväskylä, P.O. Box 35 (VIV), FIN-40014 Jyväskylä, Finland; 2 Institute for Molecular Medicine Finland (FIMM), University of Helsinki, Helsinki, Finland; 3 Department of Social Research, University of Helsinki, Helsinki, Finland; 4 Department of Public Health, University of Helsinki, Helsinki, Finland

**Keywords:** Epigenetic clock, Telomeres, Methylation, Twin design, Post-menopausal, Physical function

## Abstract

**Background:**

Biomarkers of biological aging – DNA methylation age (DNAm age) and leukocyte telomere length (LTL)– correlate strongly with chronological age across the life course. It is, however, unclear how these measures of cellular wear and tear are associated with muscle strength and functional capacity, which are known to decline with older age and are associated with mortality. We investigated if DNAm age and LTL were associated with body composition and physical functioning by examining 48 monozygotic twin sisters.

**Methods:**

White blood cell DNAm age (predicted years) was calculated from Illumina 450 k BeadChip methylation data using an online calculator. DNAm age acceleration was defined from the residuals derived from a linear regression model of DNAm age on chronological age. LTL was measured by qPCR. Total body percentage of fat and lean mass were estimated using bioimpedance. Physical functioning was measured by grip strength, knee extension strength and by 10 m maximal walking speed test.

**Results:**

In all participants, DNAm age (58.4 ± 6.6) was lower than chronological age (61.3 ± 5.9 years). Pairwise correlations of monozygotic co-twins were high for DNAm age (0.88, 95% CI 0.79, 0.97), age acceleration (0.68, 95% CI 0.30, 0.85) and LTL (0.77, 95% CI 0.60, 0.94). Increased age acceleration i.e. faster epigenetic aging compared to chronological age was associated with lower grip strength (β = − 5.3 SE 1.9 *p* = 0.011), but not with other measures of physical functioning or body composition. LTL was not associated with body composition or physical functioning.

**Conclusions:**

To conclude, accelerated DNAm age is associated with lower grip strength, a biomarker known to be associated with physiological aging, and which predicts decline in physical functioning and mortality. Further studies may clarify whether epigenetic aging explains the decline in muscle strength with aging or whether DNAm age just illustrates the progress of aging.

**Electronic supplementary material:**

The online version of this article (10.1186/s12877-018-0775-6) contains supplementary material, which is available to authorized users.

## Background

**Fig. 1:**
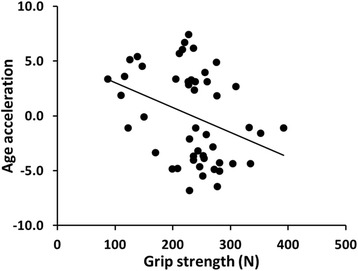
Association between grip strength and DNA methylation age acceleration in the total group of subjects (n = 48)

Many theories have emerged to explain the processes or mechanisms driving aging. It is well known that aging and especially longevity has a genetic component [1], and some environmental factors such as infections, diet, alcohol use, smoking and work exposures predispose to age-related diseases and increase probability of death. However, only a small fraction of individual variation to life expectancy can be accounted for using known and measured characteristics and exposure. Biological clocks may increase our understanding on human aging [2]. DNA methylation (DNAm) age [3] and telomere length [4] are timely and interesting biological clocks, which may provide insights into the mechanisms behind why some individuals age faster than others and are more prone to age-related diseases and accelerated decline in physical functioning (Fig. 1).

Epigenetic changes affect the function of DNA without altering the genetic code itself. Epigenetic regulation modifies the function of genetic information by controlling when specific genes are switched on or off, for example during development or aging. DNAm is one type of epigenetic modification, which changes over time. The most promising estimate for biological age developed from DNAm data is Steve Horvath’s DNAm age, also known as “Epigenetic clock” [3]. DNAm age is an age estimate based on DNA methylation at 353 specific CpG sites. DNAm age increases over chronological age, but it is not yet clear if it is only a marker of biological aging or has an effect on aging per se [3].

Telomeres are short DNA sequences located at the end of the eukaryotic chromosomes protecting functional DNA. In most somatic cells, such as in leukocytes, telomerase activity is low and telomeres shorten with every cell cycle. This is due to the inability of DNA polymerase to fully replicate chromosome ends leading to replicative senescence and apoptosis [5]. Oxidative stress enhances telomere erosion with cell replication [6], whereas inflammation entails an increase turnover of leucocytes [7]. Telomere length can be used as an indicator of cells replicative history and regenerative potential and, therefore, it might serve as an indicator of biological age [8].

Both DNAm age and telomere length are highly heritable [3, 9]. Few studies done so far have suggested that these biological age estimates are uncorrelated and therefore appear to be tagging different aspects of the aging process [2]. Faster running clock i.e. shorter telomeres or higher DNAm age compared to chronological age are associated with declining physical function with aging [10–12], and predict higher mortality risk [2, 13].

Of several age-related physiological changes, decline in muscle mass and strength are the most important factors in regards to physical functioning and quality of life. Muscle strength loss with aging together with adverse changes in body composition predispose to several age-related diseases and to decline in physical functioning, mobility and loss of independence. However, muscle strength and mass are well-preserved in some individuals up till old age, while others suffer from accelerated decline during aging. The purpose of the present study was to investigate if novel biological clocks (DNAm age and LTL) can explain interindividual variation in body composition and physical functioning in 54 to 72 year-old women.

## Methods

### Participants and study design

The participants originate from two studies: 1) “Sarcopenia - Skeletal Muscle Adaptation to Postmenopausal Hypogonadism and Effects of Hormone Replacement Therapy and Physical Activity in Older Women: a Genetic and Molecular Biological Study on Estrogen-related Pathways” (SAWEs, *n* = 15 pairs) and the Finnish Twin Study on Aging (FITSA, *n* = 9 pairs). Participants of both these studies were recruited from the Older Finnish Twin Cohort [14, 15] and the recruitment process has been described in detail elsewhere [16, 17]. Briefly, SAWEs twins were recruited for laboratory visit based on discordance for long term hormone replacement therapy (HRT), while FITSA participants participated on laboratory visit within study investigating the role of genetic and environmental factors in the disablement process in old age and HRT discordant pairs were selected from larger population afterwards. Altogether 24 monozygotic Caucasian twin pairs discordant for long term HRT were investigated. HRT users and their co-twins did not differ in regards to biological clocks, body composition or physical functioning (Additional file 1: Table S1). Twins were similar in their physical activity habits, daily energy intake, use of medication, and smoking behavior [17, 18].

### DNA methylation age and age acceleration

High molecular weight white blood cell DNA was extracted using QIAamp DNA Mini Kit (QIAGEN, Nordic, Sollentuna, Sweden). Bisulfite conversion of DNA was completed using EZ-96 DNA methylation-Gold Kit (Zymo Research, Irvine, CA, USA) according to the manufacturer’s instructions, and the co-twins were always converted on the same plate to minimize potential batch effects. Genome-wide DNAm was measured using Illumina’s Infinium HumanMethylation450 BeadChip according to the manufacturer’s instructions. The Illumina BeadChips measure single-CpG resolution DNAm levels at 485577 CpG sites across the human genome. DNAm age was calculated from 353 specific CpG-sites known to be associated with aging based on their methylation using validated algorithm and online tool [3]. Age acceleration, which describes the difference between chronological age and DNAm age (“faster or slower biological aging”) was calculated for all subjects as the residuals from a linear regression model of DNAm age on chronological age.

### Telomere length measurements

Quantitative real-time polymerase chain reaction (qPCR) based method was used determine relative LTL from peripheral blood DNA [12]. Details of the analysis as well as quality control procedures have been reported earlier [12, 19]. Briefly, a separate qPCR reaction was performed with telomere sequence-specific primers and a single copy control gene, *β-hemoglobin (HBB)*. Quality control and calculation of the T/S (telomere to single-copy gene intensity) ratios for the samples to obtain the relative telomere length was performed with Bio-Rad CFX Manager v.1.6 software. The coefficient of variation for repeated measures was 6.37% for the telomere reaction, 4.99% for the *HBB* reaction, and 6.97% for the ratio (T/S). Telomere length measurement was available from 45 of the 48 twin sisters.

### Body composition

*Body mass index (BMI)* was calculated from measured body weight and height (kg/m^2^). *Total body percentage of fat and lean mass* (LM) were estimated using bioimpedance (Spectrum II, RJL Systems, Detroit, MI, USA).

### Physical functioning

*Grip strength (N)* was measured using an isometric dynamometer (Good Strength IGS01, Metitur Oy, Jyväskylä, Finland). Best attempt of three maximal trials was used in the analysis.

*Knee extension strength (N)* was measured using an adjustable dynamometer chair (Good Strength, Metitur, Palokka, Finland). Knee extension strength was measured at the knee angle of 60° from full extension with the ankle fastened by a belt to a strain-gauge system. After familiarization, three to five maximal efforts, separated by a 1-min rest, were conducted. For each subject, the best performance with the highest value was accepted as the result.

*Maximal walking speed* over 10 m was recorded with photocells placed 71 cm from ground level and using a Digitest-1000 amplifier-time measurement system (Digi Test-1000; Digitest Ltd., Muurame, Finland). The start position was 50 cm from the photocell line and the endpoint was 10 m away from the first photocell line [17].

### Statistics

Data are shown as means and standard deviations unless otherwise stated. Intraclass correlation coefficients (ICC) were computed for the twin pairs to estimate the level of within-pair similarity. Associations between chronological age, DNAm age, age acceleration and LTL (adjusted for age) were analyzed using standardized regression coefficients to represent bivariate correlations. Age adjusted associations between biological clocks and body composition and physical functioning were tested with regression analyses. Within-pair dependency of twin individuals was taken into account using the cluster option in the analyses. HRT was also considered as a potential confounder, but it had no effect to the models (data not shown). The level of significance was set at *p* ≤ 0.05. Data analyses were carried out with R version 3.2.2 with RStudio version 0.98.932 (R Core Team 2015).

## Results

### Participants’ characteristics

The mean age of the participants (61.3 ± 5.9 years) was slightly higher than predicted by DNAm age (58.4 ± 6.8 years) (Table 1).

**Table 1:** Subject characteristics

Variable	Mean (SD)
Age	61.3 (5.9)
Biological clocks	
DNAm age (predicted years)	58.4 (6.8)
Age acceleration	0.00 (4.2)
Leukocyte telomere length	0.91 (0.15)
Body composition	
Percentage of fat (%)	32.6 (7.7)
Lean mass (kg)	47.8 (3.8)
Physical functioning	
Grip strength (N)	233 (64)
Knee extension strength (N)	389 (91)
Walking speed 10 m (s)	6.5 (1.0)

*SD* standard deviation, *DNAm* DNA methylation

*Within-pair analysis* showed that the twin sisters were highly similar for their DNAm age, age acceleration, and LTL (*r* = 0.88, 95% CI 0.79–0.97; *r* = 0.68, 0.39–0.85; *r* = 0.77, 0.60–0.94, respectively). Moderate within-pair correlations were also observed for percentage of fat (0.64, 0.33–0.83), lean mass (0.60, 0.27–0.80) and knee extension strength (*r* = 0.62, 0.30–0.81), while weaker and non-significant associations occurred for grip strength (*r* = 0.27, − 0.024−0.67) and walking speed (*r* = 0.19,−0.23–0.55). Standardized correlation coefficient between chronological age and DNAm age was 0.79 (*p* < 0.001). However, chronological age did not correlate with LTL (*r* = − 0.17, *p* = 0.34). DNAm age acceleration was not associated with LTL, when LTL was adjusted for age (residuals) (*r* = − 0.29, *p* = 0.059).

Higher age acceleration was associated with higher total body lean mass and lower grip strength, but not with percentage of fat, knee extension strength or walking speed (Table 2, Fig. 1). LTL was not associated with body composition or physical functioning in age adjusted models (Table 3).

**Table 2:** Associations between body composition, physical functioning and age acceleration in all subjects (*n* = 48)

	*Coefficients*			*Model*	95%CI	
	β	*s.e.*	*p*	*R* ^*2*^	lower	upper
Body composition						
Lean mass (kg)	0.335	0.15	0.041	0.137	0.033	0.638
Fat percent (%)	0.376	0.30	0.225	0.042	−0.214	0.967
Physical performance						
Hand grip strength (Nm)	−5.324	1.91	0.011	0.121	−9.076	−1.573
Walking speed 10 m (s)	−0.029	0.03	0.397	0.016	−0.096	0.037
Knee extension (Nm)	−3.234	3.36	0.346	0.022	−9.817	3.349

All *p*-values are assessed with linear regression

**Table 3:** Associations between body composition, physical functioning and leukocyte telomere length age in all subjects (*n* = 45)

	*Coefficients*			*Model* ^a^	95%CI	
	β	*s.e.*	*p*	*R* ^*2*^	lower	upper
Body composition						
Lean mass (kg)	−4.533	4.27	0.301	0.032	−12.906	3.841
Fat percent (%)	−10.406	12.10	0.400	0.040	−34.115	13.302
Physical performance						
Hand grip strength (Nm)	69.630	64.05	0.290	0.045	−55.912	195.164
Walking speed 10 m (s)	−0.917	0.75	0.237	0.147	−2.391	0.558
Knee extension (Nm)	−18.794	74.63	0.804	0.215	− 165.071	127.482

All p-values are assessed with linear regression. ^a^ Model fit statistics are shown for the covariate and adjusting covariate (age). s.e., standard error

## Discussion

Biological aging process is dependent on our inherited genetic background and shaped by multiple environmental, social and lifestyle factors leading to great diversity in life span, and older age health and physical functioning. This diversity within countries is found even in those well-developed, peaceful and wealthy societies that have minimized many external agents affecting average lifespan such as infections, poor living conditions, poverty and unstable civil society. These latter factors account for much of the average differences between countries globally. Even in less-developed countries, the majority of morbidity comes from non-communicable diseases, which have many common determinants (such as tobacco use, alcohol, diet and physical inactivity). Many of these common proximal factors have more distal psychological and social determinants, indicating a complex network of causation superimposed on the natural biology of our species for development and ageing.

Biological clocks may give insights into why some individuals age at faster rates compared to others, and are therefore more prone to adverse effects of aging, such as loss of muscle strength and mass, which further leads to loss of physical functioning and increased incidence of diseases. This study compared two widely used biological aging clocks, and their associations with traditional measures of physiological aging; muscle mass and physical function. The results suggest strong familial component to both LTL and DNAm age estimates, possibly due to strong genetic effects. Our study shows that the novel epigenetic aging marker, DNAm age, compared to LTL, holds more potential in tracking individual variation in physical function with aging.

Multiple biomarkers have been tested to evaluate and to predict individual aging. Shorter LTL compared to mean LTL length in cohorts at certain chronological age predicts morbidity and mortality [20]. Also higher DNAm age compared to chronological age predicts mortality risk [2, 13]. Despite these similarities, recent evidence suggests that DNAm age estimate describes cellular aging mechanism that is independent of DNA damage induced senescence and telomere length [21]. Our results support these findings showing no significant associations between LTL and Horvath’s DNAm age acceleration, while a high precision with DNAm age and chronological age was observed.

The observed within-pair correlation in LTL among monozygotic twins pairs agree with earlier findings [9, 22] suggesting strong effect of familial factors. In general, earlier studies have reported at most weak associations between LTL and physical function [23, 24]. Despite including multiple different measures of physical functioning in the current study, we also did not observe cross-sectional associations between LTL and physical functioning. Although it must be noted that our sample size is limited, the model parametric do not support existence of associations. However, it is possible that LTL predicts future development of adverse aging effects. We have recently shown that LTL predicts loss of physical function during an 11-year follow-up in older women [12], and that LTL is causally associated with cognitive function [25].

Muscle strength reaches its peak at age of 20 to 30 years and declines slowly thereafter [26]. After 50’s muscle strength loss accelerates leading to walking limitations, loss of function and increased disabilities and morbidity. Although strength loss is a universal aging effect, both peak strength level and strength decline are shaped by genetic background and multiple environmental factors leading to great diversity in physical functioning in older age. DNAm age may offer fresh perspectives into mechanisms that lead to this individual variation, but current evidence is limited [10]. In line with a previous study [10], we found that individuals with faster biological aging have lower grip strength. It is possible that an unknown cellular mechanism behind aging is affected by the biological aging markers as multiple studies have shown that grip strength has a very high predictive value in terms of incidence of functional limitations, morbidity and mortality in later life [26–28]. However, longitudinal and mechanistic studies are needed to elucidate and characterize potential causal mechanisms. Age acceleration was not associated with knee extension strength and walking speed. These measures of physical function include larger inter-individual variation and are affected by multiple confounders, such as body size and physical activity, and therefore do not reflect physiological aging as purely as decline in grip strength.

Twin studies have confirmed that the effect of familial factors is large in physical functioning and body lean mass. Large scale genome-wide association studies have not been published for many traits related to physical functioning, and only a minor fraction of underlying genetic factors has been attributed to specific genes or genotypes [29]. The genetic architecture of body composition is better known, in particular of height, overall BMI and regional adiposity [30, 31], and much of their heritability is accounted for identified genes [32]. It is not known, what is the role of epigenetic factors, such as DNAm, in this “hidden heritability” [33]. This study revealed minor or non-existent associations between body composition and DNAm age acceleration. We found a positive association between DNAm age acceleration and total body lean mass. This result may be partly explained by the fact that body fat and lean mass at the total body level often increases slowly over adult age, even though muscle mass in lower extremities often decreases over adult age, similarly as muscle strength. Another explanation for this finding is rather narrow distribution in lean mass among the study sample, as subjects with severe morbidity, sarcopenia or frailty were not included in the study. In addition, our study subjects represent a sample of rather young same-sex individuals.

Studies on body composition and telomere length have relied almost exclusively on indirect anthropometric measures such as BMI [34]. We used the bioimpedance method for establishing body composition, and were able to test for associations of percentage of fat and lean mass with biological aging separately, but did not observe any significant associations. It has been speculated that body composition and especially obesity may affect telomere attrition through multiple mechanisms, but studies have reported very inconsistent results [34]. Most likely associations between telomere shortening in adulthood and old age has very minor effects in poorly proliferating tissues such as fat or muscle tissue.

In this study aging biomarkers were analyzed using blood samples rather than muscle tissue, which may be more relevant tissue in terms of physical function. Telomere length varies depending on the sample origin and telomeres from different tissues may have divergent associations with the amount and distribution of body fat and muscle mass [19]. Also different tissues in the same individual may vary in terms of their DNAm age [3]. Aging of the liver, rather than blood, muscle or fat tissue, is accelerated in obese subjects [35]. Muscle tissue is the key tissue affecting age-related decline in physical functioning, several age-related diseases such as type II diabetes and cardiovascular diseases, and even mortality [36, 37]. At least two studies have reported modest to high correlations between DNAm age in muscle tissue and chronological age [3, 35]. However, none of these studies have investigated DNAm age of the muscle tissue in association with physical functioning. Future studies examining DNAm age in muscle tissue may hold potential for identifying genes, pathways, and regulatory mechanisms of importance for disease development and age-related decline in physical functioning.

Monozygotic female twin pairs were used in this study, resulting in limited sample size, which in turn may reduce the credibility when generalizing our results to general population. The ICCs in monozygotic twin pairs estimate the proportion of familial factors in the variables under investigation. However, these correlations provide only an upper limit to the heritability although the relative roles of genetic and shared environmental factors cannot be teased apart. The strength of our study was that physical function measurements were performed by experienced research personnel and in standardized laboratory settings. Some criticism has been leveled about rationale and measurements of both aging biomarkers that were investigated in this study. Quantitative real-time PCR method used in this study to measure LTL has been validated and is widely used [38]. Although DNAm age is a relatively new biomarker of aging, and it is not known what it really captures [3, 35], it has been shown to correlate highly with chronological age and to be associated with multiple aging related phenotypes and diseases [10, 35]. However, there are several estimates for quantification of biological age from DNAm data, and some of these may be more consistently related to phenotypes related to physical functioning [39].

## Conclusions

This study support earlier findings that DNAm is associated with biological aging [10]. DNAm age acceleration seems to share similar individual age trajectories with grip strength, which is a strong predictor of development of older age disabilities and mortality [26–28, 40]. Further studies are required to establish whether DNAm regulates aging pathways related to physical function or whether it is just a biomarker that correlates highly with grip strength and chronological age.

## Additional file

Additional file 1: **Table S1.** Differences between users and non-users from 24 twin pairs discordant for hormone replacement therapy. (DOCX 21 kb)

## Abbreviations

BMI: Body mass index
DNAm age: DNA methylation age
FITSA: the Finnish Twin Study on Aging
HBB: β-hemoglobin
HRT: hormone replacement therapy
ICC: Intraclass correlation coefficient
LM: body lean mass
LTL: leukocyte telomere length
qPCR: quantitative real-time polymerase chain reaction
SAWEs: Skeletal Muscle Adaptation to Postmenopausal Hypogonadism and Effects of Hormone Replacement Therapy and Physical Activity in Older Women: a Genetic and Molecular Biological Study on Estrogen-related Pathways
