# Supplementary material for: Biological clocks and physical functioning in monozygotic female twins
Source: BMC Geriatr. 2018 Apr 4;18:83. doi: 10.1186/s12877-018-0775-6 (PMC5883300; doi:10.1186/s12877-018-0775-6)
Supplement: Supplementary file 1 — Table S1. Differences between users and non-users from 24 twin pairs discordant for hormone replacement therapy. (DOCX 21 kb) [file 12877_2018_775_MOESM1_ESM.docx]

**Additional file 1: Table S1.** Differences between users and non-users from 24 twin pairs discordant for hormone replacement therapy.

|  |  | HRT users | HRT non-users | Between groups |
| --- | --- | --- | --- | --- |
|  |  | Mean (SD) | Mean (SD) | p |
|  |  |  |  |  |
| Chronological age (years) | | 61 (6.0) | 61 (6.0) |  |
| Biological clocks | |  |  |  |
|  | DNAm age (predicted years) | 58.5 (7.3) | 58.4 (6.3) | 0.247 |
|  | Age acceleration | 0.39 (2.6) | -0.39 (2.9) | 0.247 |
|  | Leukocyte telomere length | 0.91 (0.16)* | 0.91 (0.13)# | 0.248 |
| Body composition | |  |  |  |
|  | Body mass index (kg/m^2^) | 27.3 (4.4) | 28.1 (5.7) | 0.135 |
|  | Percentage fat (%) | 31.8 (7.5) | 33.4 (8.0) | 0.071 |
|  | Body lean mass (kg) | 48.0 (3.4) | 47.5 (4.2) | 0.849 |
| Physical performenace | |  |  |  |
|  | Hand grip strength (N) | 242 (70) | 225 (57) | 0.627 |
|  | Knee extension strength (N) | 393 (84) | 385 (99) | 0.370 |
|  | Walking speed 10m (s) | 6.5 (1.0) | 6.5 (0.9)* | 0.494 |
|  |  |  |  |  |

HRT, hormone replacement therapy; SD, standard deviation; HRT users n=24; HRT non-users n=24; * n=23; # n=22; Between group difference tested by Wilcoxon signed rank test
